# Increased blood pressure variability during the subacute phase of ischemic stroke is associated with poor functional outcomes at 3 months

**DOI:** 10.1038/s41598-020-57661-z

**Published:** 2020-01-21

**Authors:** Hiroyuki Naito, Naohisa Hosomi, Daisuke Kuzume, Tomohisa Nezu, Shiro Aoki, Yuko Morimoto, Masato Kinboshi, Takeshi Yoshida, Yuji Shiga, Naoto Kinoshita, Hiroki Ueno, Kensuke Noma, Masahiro Yamasaki, Hirofumi Maruyama

**Affiliations:** 1 Department of Clinical Neuroscience and Therapeutics, Hiroshima University Graduate School of Biomedical and Health Sciences, Hiroshima, Japan; 2 Department of Neurology, Chikamori Hospital, Kochi, Japan; 3 Department of Rheumatology, Chikamori Hospital, Kochi, Japan; 4 Department of Cardiovascular Regeneration and Medicine, Research Institute for Radiation Biology and Medicine, Hiroshima University, Hiroshima, Japan

**Keywords:** Hypertension, Stroke

## Abstract

Thus far, it is well known that increased blood pressure variability may exacerbate stroke outcomes. Blood pressure in the acute phase would be influenced by both reactive hypertension to stroke and intrinsic blood pressure reactivity. Thus, we aimed to evaluate the association between blood pressure variability and outcomes at 3 months using ambulatory blood pressure monitoring in ischemic stroke patients in the subacute phase after reactive hypertension subsided. We retrospectively examined 626 consecutive patients with acute ischemic stroke who underwent 24-hour ambulatory blood pressure monitoring during the subacute phase of stroke (median, 9 days from onset). The variability in blood pressure was evaluated by assessing the standard deviation and coefficient of variation of systolic and diastolic blood pressure. The primary outcome was functional status at 3 months. A poor outcome was defined as a modified Rankin scale score of 3 or more and a good outcome as 2 or less. We assessed the functional outcome at 3 months in 497 patients (79.4%). The mean systolic and diastolic blood pressure levels were not associated with functional outcome. The multivariable analysis revealed that increases in the standard deviations of systolic and diastolic blood pressure, coefficient of variation of diastolic blood pressure, and morning blood pressure surge were associated with poor outcome. Blood pressure variability during the subacute phase of ischemic stroke can be a useful prognostic indicator of poor functional outcome at 3 months in patients with acute ischemic stroke.

## Introduction

Hypertension is a major risk factor for ischemic stroke^1^. Antihypertensive treatment in the chronic phase of stroke is recommended for the prevention of stroke^2^. Approximately 80% of patients in the acute phase of ischemic stroke have been reported to have reactive hypertension^3,4^. Several studies suggest that elevated blood pressure (BP) levels are a poor prognostic factor after acute ischemic stroke (AIS)^5,6^, while other studies could not find any association between them^7,8^. Therefore, the management of hypertension in the acute stage of ischemic stroke remains controversial.

High BP variability in the acute phase of ischemic stroke has also been reported to lead to poor outcomes because such variability during the first 72 hours of stroke onset promotes hemorrhagic transformation and increases the risk of lesion growth and recurrence^9,10^. However, a systematic review and meta-analysis found no association between BP variability during the first 24 hours of admission and stroke outcome^11^. Previous studies have explained some of these conflicting results. For instance, in the acute phase of large ischemic stroke, reactive hypertension and impaired cerebral blood flow can occur^12^, and elevated BP becomes stable during the first 4 to 7 days after stroke onset^13,14^. Therefore, BP in the acute phase would be influenced by both reactive hypertension and stroke and intrinsic BP reactivity. In addition, there are differences in the intervals between BP measurements across the various studies. Compared with casually recorded BP, ambulatory 24-hour BP monitoring (ABPM) can accurately evaluate clinical status and reduce observer bias^15,16^.

We hypothesized that increased BP variability in the subacute phase, when restored after reactive hypertension, may be associated with stroke outcomes. In this study, we aimed to evaluate the association between each BP parameter and outcomes at 3 months using ABPM in ischemic stroke patients in the subacute phase.

## Results

### Baseline characteristics and patient outcomes

Of the 626 total patients, 497 (79.4%) had their functional outcomes assessed at 3 months. The patients who were not evaluated for stroke outcomes at 3 months had a lower frequency of diabetes mellitus and previous ischemic heart disease and had a different distribution of stroke subtypes (Supplemental Table 1). Of the 497 patients who were evaluated for functional outcomes, 184 (37.0%) had poor outcomes. Baseline characteristics are shown in Table 1. The patients with poor outcomes were significantly older (p < 0.001), had a lower body mass index (BMI, p = 0.013), and exhibited a higher frequency of chronic kidney disease (CKD, p < 0.001) and atrial fibrillation (p < 0.001) than those with good outcomes; moreover, fewer of these patients were male (p = 0.027). Fewer patients with poor outcomes were smokers than those with good outcomes (p < 0.001). The patients with poor outcomes exhibited severe neurological deficits at admission (p < 0.001). The distribution of stroke subtypes was significantly different between patients with good and poor outcomes (p < 0.001). Of the 626 total patients, 204 (32.6%) reported current use of antihypertensive medication at the ABPM measurement. Among the antihypertensive drugs, there was a prevalence of Ca–blockers (96 cases), angiotensin–receptor blockers (81 cases), beta–blockers (65 cases), diuretic drugs (57 cases), alpha–blockers (8 cases), and angiotensin-converting enzyme inhibitors (7 cases).

**Table 1:** Baseline characteristics at admission and univariate analysis of factors associated with 3-month functional outcome.

	mRS 0–2 (n = 313)	mRS 3–6 (n = 184)	p
Age, years	71.0 ± 12.0	79.6 ± 8.9	<0.001
Sex, male	200 (63.9)	99 (53.8)	0.027
Body mass index, kg/m^2^	23.7 ± 3.7	22.9 ± 4.0	0.013
Daily alcohol intake	81 (25.9)	38 (20.7)	0.19
Current smoking	85 (27.2)	25 (13.6)	<0.001
Hypertension	218 (69.7)	140 (76.1)	0.12
Diabetes mellitus	106 (33.9)	62 (33.7)	0.97
Dyslipidemia	141 (45.1)	83 (45.1)	0.99
Chronic kidney disease	103 (32.9)	93 (50.5)	<0.001
Atrial fibrillation	55 (17.6)	58 (31.5)	<0.001
Previous stroke	88 (28.1)	66 (35.9)	0.07
Previous ischemic heart disease	41 (13.1)	25 (13.6)	0.88
Use of antihypertensive medication at the ABPM measurement	110 (35.1)	61 (33.2)	0.65
NIHSS score at admission	2 (1–4)	5.5 (2–12)	<0.001
Stroke subtype			<0.001
Small-vessel occlusion	65 (20.8)	18 (9.8)	
Large-artery atherosclerosis	94 (30.0)	65 (35.3)	
Cardioembolic stroke	60 (19.2)	59 (32.1)	
Other etiology	94 (30.0)	42 (22.8)	

Data are presented as the means ± standard deviation for age and body mass index, as median (interquartile range) for baseline NIHSS score, and as number of patients (%) for others.

ABPM, ambulatory 24-h BP monitoring; NIHSS, National Institutes of Health Stroke Scale; mRS, modified Rankin scale.

### BP parameters and patient outcomes

**Figure 1:**
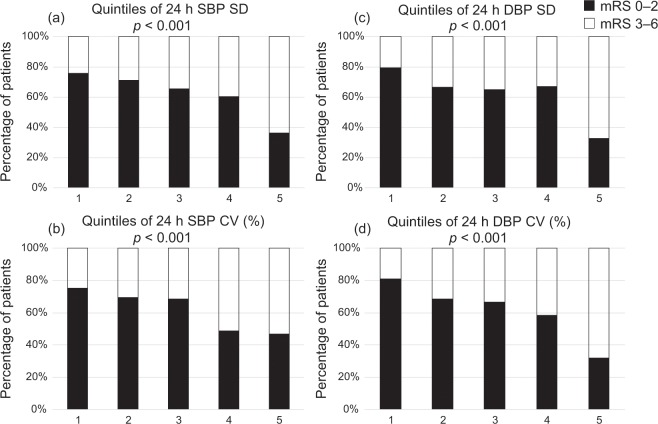
Distribution of mRS scores at 3 months according to BP parameter quintiles (**a**) SBP SD, (**b**) SBP CV (%), (**c**) DBP SD, and (**d**) DBP CV (%). The percentage of patients with an mRS score of 0–2 decreased as the BP variability increased [mRS of 0–2 (black columns), mRS of 3–6 (open columns)]. P values were calculated using the Cochran-Armitage test for trend. BP, blood pressure; mRS, modified Rankin scale; SBP, systolic blood pressure; DBP, diastolic blood pressure; SD, standard deviation; CV, coefficient of variation.

In the univariate analysis, systolic and diastolic BP (SBP and DBP) at admission and the average values of 24-hour SBP and DBP were not associated with poor outcome at 3 months (Table 2). The patients with poor outcomes had a higher frequency of morning surge and nondipper types and higher variability in all BP indicators than those with good outcomes. The analysis according to the BP parameter quintile showed that the proportion of patients with poor functional outcomes significantly increased as each quintile of BP variability increased (Fig. 1). In contrast, the average values of 24-hour SBP and DBP did not show a similar trend. After adjusting for age, sex, and the National Institutes of Health Stroke Scale (NIHSS) score at admission, standard deviations (SDs) of 24-hour SBP and DBP, coefficient of variation (CV) of 24-hour DBP, and presence of morning surge were found to be independently associated with poor outcome at 3 months (model 1, Table 3). In addition, each BP variability parameter was also associated with poor outcome after adjusting for other baseline confounding factors (model 2, Table 3 and Supplemental Table 2). In a similar analysis, the SD of 24-hour SBP and DBP, CV of 24-hour DBP, and presence of morning surge were also associated with poor outcome in the patients not using antihypertensive medication at the ABPM measurement (Supplemental Table 3). After adding the SD of 24-hour DBP to the aforementioned clinical factors (age, sex, and NIHSS score at admission), the c-statistic showed a significantly increased ability to predict the functional outcome (p = 0.048) (model 1, Table 4). However, after adding each BP parameter to the clinical factors (model 2, Table 4), the c-statistic did not demonstrate a significantly increased ability.

**Table 2:** Blood pressure parameters and univariate analysis of factors associated with 3-month functional outcome.

	mRS 0–2 (n = 313)	mRS 3–6 (n = 184)	p
SBP at admission	161.1 ± 29.4	158.5 ± 31.6	0.36
DBP at admission	90.0 ± 20.4	88.8 ± 19.3	0.51
24-hour SBP mean	128.9 ± 16.5	131.7 ± 19.0	0.08
24-hour SBP SD	15.8 ± 4.2	18.4 ± 5.1	<0.001
24-hour SBP CV	12.4 ± 3.4	14.1 ± 4.1	<0.001
24-hour DBP mean	79.1 ± 10.7	79.3 ± 12.8	0.85
24-hour DBP SD	14.2 ± 5.5	18.5 ± 8.4	<0.001
24-hour DBP CV	18.1 ± 6.9	23.3 ± 8.9	<0.001
Morning surge	53 (17.7)	53 (29.1)	0.004
Nondipper type	265 (84.7)	168 (91.3)	0.033

BP parameters are presented as the means ± standard deviation and as number of patients (%) for others.

BP, blood pressure; SBP, systolic blood pressure; DBP, diastolic blood pressure; SD, standard deviation; CV, coefficient of variation; mRS, modified Rankin scale.

**Table 3:** Indicators associated with poor outcome at 3 months.

Indicators	Model 1		Model 2	
	OR (95% CI)	p	OR (95% CI)	p
Clinical factors	—	—	—	—
24-hour SBP SD	1.39 (1.12–1.72)	0.002	1.42 (1.14–1.78)	0.002
24-hour SBP CV	1.21 (0.98–1.49)	0.08	1.23 (0.98–1.53)	0.07
24-hour DBP SD	1.54 (1.25–1.90)	<0.001	1.54 (1.24–1.91)	<0.001
24-hour DBP CV	1.56 (1.24–1.95)	<0.001	1.60 (1.27–2.03)	<0.001
Morning surge	1.81 (1.08–3.03)	0.025	1.92 (1.12–3.27)	0.017
Nondipper type	1.45 (0.73–2.87)	0.28	1.42 (0.71–2.85)	0.31

Multivariable logistic analyses were performed to identify indicators (model 1: age, sex, NIHSS score at admission, and each BP parameter using ABPM; model 2: age, sex, variables (except for stroke subtypes) with p values less than 0.20 in the univariate analysis, and each BP parameter) for poor outcome.

SBP, systolic blood pressure; DBP, diastolic blood pressure; SD, standard deviation; CV, coefficient of variation; OR, odds ratio; CI, confidence interval.

**Table 4:** C-statistic value for the prediction of poor outcome at 3 months.

Indicators	Model 1			Model 2		
	C-statistic	Change in c-statistic	p	C-statistic	Change in c-statistic	p
Clinical factors	0.802	—	Referent	0.818	—	Referent
24-hour SBP SD	0.809	0.007	0.31	0.826	0.008	0.21
24-hour SBP CV	0.805	0.003	0.48	0.820	0.002	0.59
24-hour DBP SD	0.822	0.020	0.048	0.834	0.016	0.07
24-hour DBP CV	0.819	0.017	0.07	0.833	0.015	0.07
Morning surge	0.803	0.001	0.89	0.821	0.003	0.42
Nondipper type	0.804	0.002	0.43	0.819	0.001	0.39

The c-statistic for each model (model 1: age, sex, NIHSS score at admission; model 2: age, sex, variables (except for stroke subtypes) with p values less than 0.20 in the univariate analysis) was calculated to assess each model’s ability to predict the stroke outcome. The p value for the increase in c-statistic in a model with each BP parameter added to the clinical factors was compared with that in a model with clinical factors only.

SBP, systolic blood pressure; DBP, diastolic blood pressure; SD, standard deviation; CV, coefficient of variation.

## Discussion

In the present study, we found that BP variability during the subacute phase of ischemic stroke was significantly associated with a poor 3-month functional outcome in patients with AIS, even after adjusting for age, sex, initial stroke severity, and other confounding factors. In addition, patients in the higher quintiles of BP variability exhibited proportionally increases in poor functional outcome. A morning BP surge was also independently associated with poor functional outcome after adjusting for baseline characteristics.

The association between BP variability during the acute stage of ischemic stroke and outcomes has been investigated recently, but the relationship remains unclear. Various factors, such as brain edema, hemorrhagic transformation, vessel recanalization, increased sympathetic drive, and mental stress at admission, affect acute-phase BP levels^17–20^. In addition, reactive hypertension occurs after the onset of stroke, and elevated BP levels fall remarkably during the first 7 days of onset, with little change thereafter^21^. Thus, we aimed to evaluate the association of BP variability during the subacute phase after reactive hypertension subsides with 3-month outcomes.

Only a few reports on the relationship between BP variability during the subacute stage of AIS and stroke outcomes are available, in contrast to several reports on BP fluctuation in the acute phase. Previous reports suggested that BP variability during the subacute stage was associated with functional outcome at 3 months after stroke onset, independent of mean BP level^22,23^. In the present study, BP variability, but not average BP, during the subacute stage of ischemic stroke was significantly associated with poor outcomes at 3 months in the multivariable analysis, as previously reported^22^. Higher SBP and DBP variability quintiles were also proportionally associated with poor outcomes at 3 months. DBP was a more useful predictor of poor functional outcome than other BP parameters after analysis of the c-statistic. A previous report showed that increased DBP rather than SBP variability during the acute phase of stroke was independently associated with worse functional outcome at 3 months^24^. In contrast, another study showed that greater SBP variability during the acute phase is a significant predictor of poor functional outcome at 3 months^25^. Thus, further investigation is needed to determine whether SBP or DBP has a greater effect on outcomes.

There are several causes for the association between BP variability during the subacute phase and stroke outcome. First, there are complications, such as hyperthermia, urinary infections, hypertension, and hypoxia^26^, that may occur during the subacute phase and influence BP variability and subsequent stroke outcome. Second, BP transition may differ according to the severity of the neurological deficits in AIS^14^. Recovery from a high BP state was observed during the first 4 days after admission in patients with mild stroke and was associated with a favorable outcome, whereas a persistently high BP beyond the first 7 days was associated with severe stroke and poor outcome. Although the detailed clinical courses in the acute phase (within 7 days of stroke onset) could not be collected in this study, several factors, including neurological deterioration or infection in the acute phase, might cause BP variability in the subacute phase. Third, impaired cerebral autoregulation may be present in the subacute stage of AIS^27–29^. Because BP variability is linked to cerebral autoregulation, increased BP variability may affect the hypoperfused brain. Kang *et al*. speculated that BP variability during the subacute phase of ischemic stroke puts patients at risk of stroke recurrence and other vascular events^22^.

Our study also used 24-hour ABPM to investigate the association between the morning BP surge and functional stroke outcome. The morning surge represents BP fluctuations specific to the early morning and is an important risk factor for cardiovascular events. There is accumulating evidence suggesting a significant association between morning BP surge and cardiac, cerebral, renal, and vascular damage^30^. In our study, a higher morning BP surge was associated with poor functional outcomes in multivariable analysis. To our knowledge, there are no previous reports describing a relationship between morning BP surge during the subacute stage of AIS and stroke outcome. This finding underscores the importance of paying attention to elevated BP after waking in patients in the subacute stage of stroke.

Our study has several limitations. First, this was a retrospective double-center study. However, the baseline characteristics of the patients in this study were not remarkably different from those previously reported in a large Japanese stroke registry study^31^. Second, because it is a retrospective study, the ABPM data evaluated did not comply with the strictly determined rules. BP can be affected by neurological status, such as level of consciousness or severity of paralysis, and the patients’ activity, including bedded state and gait in wheel-chair or with a cane, during the measurement of ABPM. The severity of the neurological status at admission was assessed by the NIHSS score. However, this study enrolled hospitalized patients with AIS, and thus, participants were more likely to be at rest in the hospital than at home, taking into account the possibility that BP fluctuations were more alleviated depending on the patients’ conditions. In addition, the use of antihypertensive medications during the subacute ischemic phase was dependent on the decisions of attending physicians. In the evaluation of ABPM, the use of antihypertensive medications was not associated with stroke outcome in a univariate analysis. We also evaluated the relationship between BP variability and stroke outcome in patients without the use of antihypertensive medications, which showed similar results. A total of 34.4% of the patients had taken antihypertensive drugs. In this study, ABPM was performed a median of 9 days from stroke onset, and there were many cases in which antihypertensive agents had not yet been administered despite the patient having a high BP. Although the effects of antihypertensive drugs were considered, the results of the analysis of the antihypertensive agent-free group were similar to those of the overall subject group. Therefore, the effect of antihypertensive agents on the association between BP fluctuation and functional stroke outcome may have been limited. In a previous report, reducing BP variability using antihypertensive medications may have led to better outcomes in the chronic phase of ischemic stroke^32^. Further prospective studies are needed to determine whether active antihypertensive management for reducing BP variability during the subacute ischemic phase contributes to good outcomes.

In conclusion, BP variability during the subacute phase of ischemic stroke can be a useful prognostic indicator of poor functional outcome at 3 months in patients with AIS.

## Methods

### Study population

This was a double-center, hospital-based retrospective study involving consecutive patients with AIS hospitalized in the Hiroshima University Hospital and Chikamori Hospital. We excluded patients who were treated with intravenous thrombolysis or endovascular therapy in the present study. We enrolled a consecutive series of 840 patients hospitalized for AIS who underwent 24-hour ABPM during the subacute phase of stroke (median 9 days from onset) between April 2010 and March 2018. Of 840 patients, 214 were excluded because their premorbid modified Rankin scale (mRS) score was 3 or higher. This study was performed under the opt-out method, as it was performed retrospectively using clinical records. Informed consent for participation was not obtained from the participants.

### Assessment of clinical characteristics

The following clinical characteristics were recorded at admission: age, sex, BMI, and classical vascular risk factors including hypertension, diabetes mellitus, dyslipidemia, CKD, atrial fibrillation, daily alcohol intake (>40 g), smoking habit (current smokers or non–current smokers), and history of stroke and ischemic heart disease. Criteria for hypertension, diabetes mellitus, dyslipidemia, and atrial fibrillation were previously defined^33^. CKD was defined as an estimated glomerular filtration rate <60 mL/min per 1.73 m^2^ ^34^. The use of antihypertensive medication at the ABPM measurement was evaluated from the medical records. AIS was defined as the sudden onset of acute neurologic deficits, confirmed by imaging of brain computed tomography or magnetic resonance imaging, and patients with AIS were hospitalized within 7 days of onset. The severity of the neurological status was assessed according to the NIHSS score. Stroke subtypes were classified as small-vessel occlusion, large-artery atherosclerosis, cardioembolic stroke, and other etiology according to the Trial of ORG 10172 in Acute Stroke Treatment classification^35^.

### Assessments of BP variability

To avoid the influence of reactive hypertension after stroke, 24-hour ABPM (FB-270, Fukuda Denshi, Tokyo, Japan) was performed during the subacute stage of stroke (median 9 days from stroke onset, interquartile range 8–10). The distribution of the number of patients evaluated for ABPM according to days after stroke onset is shown in Supplemental Fig. 1. SBP and DBP were autonomically measured every 30 minutes for 24 hours. We used the raw data to obtain 24-hour mean SBP/DBP, and we calculated the SD and CV ([%]; = SD × 100/mean value) during the 24-hour period. The sleep–trough morning BP surge was calculated as the mean SBP during the 2 hours after waking minus the lowest SBP during sleep and was defined as an increase in SBP ≥ 55 mmHg from the lowest SBP during sleep^36^. Patients were classified into two groups according to their decrease (%) in mean SBP during the nighttime compared with the daytime: dipper (fall ≥ 10%) and nondipper (fall < 10%)^37^.

### Outcome assessment

The primary outcome evaluated was the 3-month functional status. A poor outcome was defined as an mRS score of 3–6, and a good outcome as an mRS score of 0–2.

### Statistical analysis

Categorical variables are presented as numbers and percentages, and continuous variables are presented as the means with SD or median (interquartile range). Comparisons of baseline characteristics between patients with good and poor outcomes were performed using the χ^2^ test for categorical variables and Student’s t-test or the Mann-Whitney U test for continuous variables. The Cochran-Armitage test for trend was utilized to examine changes according to BP parameters classified into quintiles. Multivariable logistic regression analyses were performed to identify indicators (model 1: age, sex, NIHSS score at admission, and each BP parameter using ABPM; model 2: age, sex, variables (except for stroke subtypes) with p values less than 0.20 in the univariate analysis, and each BP parameter) for poor outcome. We calculated the c-statistic for models with age, sex, and NIHSS score at admission to assess each model’s ability to predict the stroke outcome. Adding each BP parameter to those indicators, the changes in the c-statistic were also analyzed as previously described^38^. In all analyses, p < 0.05 was considered statistically significant. All analyses were performed using JMP 14.0 (SAS Institute, Inc., Cary, NC).

The data that supported the findings of this study are available from the corresponding author upon reasonable request.

### Ethics approval

This study complies with the Declaration of Helsinki for investigations involving humans, and the study protocol was approved by the Ethics Committees of Hiroshima University (#E-978) and Chikamori Hospital (#222). We disclosed the information about this study in the opt-out form.

## Supplementary information

Supplementary information .
