# Supplementary material for: Increased blood pressure variability during the subacute phase of ischemic stroke is associated with poor functional outcomes at 3 months
Source: Sci Rep. 2020 Jan 21;10:811. doi: 10.1038/s41598-020-57661-z (PMC6972830; doi:10.1038/s41598-020-57661-z)
Supplement: Supplementary file 1 — Supplementary information . [file 41598_2020_57661_MOESM1_ESM.docx]

**Supplementary Information**

**Title**

**Increased blood pressure variability during the subacute phase of ischemic stroke is associated with poor functional outcomes at 3 months**

**Authors:**

Hiroyuki Naito, MD, PhD^1^; Naohisa Hosomi, MD, PhD^1*^; Daisuke Kuzume, MD^2^; Tomohisa Nezu, MD, PhD^1^; Shiro Aoki, MD, PhD^1^; Yuko Morimoto, MD^2^; Masato Kinboshi, MD^2^; Takeshi Yoshida, MD^3^; Yuji Shiga, MD^1^; Naoto Kinoshita, MD^1^; Hiroki Ueno, MD, PhD^1^; Kensuke Noma, MD, PhD^4^; Masahiro Yamasaki, MD^2^; Hirofumi Maruyama, MD, PhD^1^

1. Department of Clinical Neuroscience and Therapeutics, Hiroshima University Graduate School of Biomedical and Health Sciences, Hiroshima, Japan
2. Department of Neurology, Chikamori Hospital, Kochi, Japan
3. Department of Rheumatology, Chikamori Hospital, Kochi, Japan

4. Department of Cardiovascular Regeneration and Medicine, Research Institute for

Radiation Biology and Medicine, Hiroshima University, Hiroshima, Japan

To whom all correspondence should be addressed: nhosomi@hiroshima-u.ac.jp

**Supplemental Figure 1.** The distribution of numbers of patients evaluated for ABPM according to days after stroke onset

**Supplemental Table 1.** Differences in baseline characteristics between the study cohort and patients excluded due to a lack of functional outcome assessment at 3 months

|  | Patients in this study (n = 497) | Patients excluded due to a lack of functional outcome assessment  at 3 months (n = 129) | p |
| --- | --- | --- | --- |
| Age (years) | 74.2 ± 11.7 | 74.6 ± 11.6 | 0.75 |
| Sex, male | 299 (60.2) | 70 (54.3) | 0.23 |
| Body mass index, kg/m^2^ | 23.4 ± 3.9 | 23.2 ± 4.0 | 0.59 |
| Daily alcohol intake | 119 (23.9) | 35 (27.1) | 0.45 |
| Current smoking | 110 (22.1) | 28 (21.7) | 0.92 |
| Hypertension | 358 (72.0) | 84 (65.1) | 0.13 |
| Diabetes mellitus | 168 (33.8) | 26 (20.2) | 0.003 |
| Dyslipidemia | 224 (45.1) | 60 (46.5) | 0.77 |
| Chronic kidney disease | 196 (39.4) | 43 (33.3) | 0.20 |
| Atrial fibrillation | 113 (22.7) | 22 (17.1) | 0.16 |
| Previous stroke | 154 (31.0) | 42 (32.6) | 0.73 |
| Previous ischemic heart disease | 66 (13.3) | 8 (6.2) | 0.027 |
| Use of antihypertensive medication at the ABPM measurement | 171 (34.4) | 33 (25.6) | 0.057 |
| NIHSS score at admission | 3 (1–6) | 2 (1–4) | 0.32 |
| Stroke subtype |  |  | 0.032 |
| Small-vessel occlusion | 83 (16.7) | 33 (25.6) |  |
| Large-artery atherosclerosis | 159 (32.0) | 29 (22.5) |  |
| Cardioembolic stroke | 119 (23.9) | 26 (20.2) |  |
| Other etiology | 136 (27.4) | 41 (31.8) |  |

Data are presented as the means ± standard deviation for age and body mass index, as median (interquartile range) for baseline NIHSS score, and as number of patients (%) for others.

ABPM, ambulatory 24­-h BP monitoring; NIHSS, National Institutes of Health Stroke Scale

**Supplemental Table 2.** Indicators associated with poor outcome at 3 months

|  | OR | 95% CI | OR | 95% CI | OR | 95% CI | OR | 95% CI | OR | 95% CI | OR | 95% CI |
| --- | --- | --- | --- | --- | --- | --- | --- | --- | --- | --- | --- | --- |
| Age (years) | 1.06^＊^ | 1.04–1.09 | 1.07^＊^ | 1.04–1.09 | 1.06^＊^ | 1.04–1.09 | 1.05^＊^ | 1.03–1.08 | 1.07^＊^ | 1.04­–1.10 | 1.07^＊^ | 1.04–1.10 |
| Sex, male | 0.99 | 0.61–1.63 | 1.01 | 0.62–1.65 | 0.91 | 0.55–1.50 | 0.98 | 0.59–1.61 | 0.98 | 0.59–1.62 | 1.00 | 0.61–1.64 |
| Body mass index, kg/m^2^ | 0.98 | 0.92–1.05 | 0.98 | 0.92–1.05 | 1.00 | 0.94–1.07 | 1.00 | 0.94–1.07 | 0.99 | 0.93–1.06 | 0.98 | 0.92–1.05 |
| Daily alcohol intake | 1.49 | 0.84–2.64 | 1.54 | 0.88–2.72 | 1.54 | 0.86–2.74 | 1.57 | 0.88–2.80 | 1.62 | 0.91–2.90 | ­1.55 | 0.88–2.74 |
| Current smoking | 0.58 | 0.32­–1.07 | 0.59 | 0.32–1.08 | 0.56 | 0.30–1.04 | 0.53^＊^ | 0.28–0.97 | 0.59 | 0.32–1.09 | 0.59 | 0.32–1.08 |
| Hypertension | 1.01 | 0.60–1.71 | 1.07 | 0.63–1.80 | 1.04 | 0.61–1.77 | 1.14 | 0.67­–1.93 | 0.92 | 0.54–1.56 | 1.02 | 0.61–1.71 |
| Chronic kidney disease | 1.56 | 0.98–2.50 | 1.58 | 0.99–2.52 | 1.43 | 0.89–2.30 | 1.44 | 0.90–2.32 | 1.72^＊^ | 1.07–2.76 | 1.58 | 0.99–2.51 |
| Atrial fibrillation | 1.01 | 0.58–1.74 | 0.96 | 0.56–1.65 | 1.01 | 0.58–1.74 | 0.97 | 0.56–1.68 | 1.01 | 0.59–1.75 | 1.01 | 0.59–1.73 |
| Previous stroke | 1.69^＊^ | 1.06–2.70 | 1.63^＊^ | 1.02–2.59 | 1.67^＊^ | 1.04–2.68 | 1.69^＊^ | 1.05–2.70 | 1.64^＊^ | 1.02–2.63 | 1.62^＊^ | 1.02–2.57 |
| NIHSS score at admission | 1.21^＊^ | 1.15–1.28 | 1.21^＊^ | 1.15–1.28 | 1.21^＊^ | 1.14–1.27 | 1.21^＊^ | 1.14–1.27 | 1.22^＊^ | 1.15–1.28 | 1.21^＊^ | 1.15­–1.28 |
| 24-h SBP SD | 1.42^＊^ | 1.14–1.78 | － | － | － | － | － | － | － | － | － | － |
| 24-h SBP CV | － | － | 1.23 | 0.98–1.53 | － | － | － | － | － | － | － | － |
| 24-h DBP SD | － | － | － | － | 1.54^＊^ | 1.24–1.91 | － | － | － | － | － | － |
| 24-h DBP CV | － | － | － | － | － | － | 1.60^＊^ | 1.27–2.03 | － | － | － | － |
| Morning surge | － | － | － | － | － | － | － | － | 1.92^＊^ | 1.12–3.27 | － | － |
| Nondipper type | － | － | － | － | － | － | － | － | － | － | 1.42 | 0.71–2.85 |

Multivariable logistic regression analyses were performed to identify indicators (age, sex, variables (except for stroke subtypes) with p values less than 0.20 in the univariate analysis and each BP parameter) for poor outcome. *p<0.05

NIHSS, National Institutes of Health Stroke Scale; SBP, systolic blood pressure; SD, standard deviation; CV, coefficient of variation; DBP, diastolic blood pressure; OR: odds ratio; CI, confidence interval.

**Supplemental Table 3.** Indicators associated with poor outcome at 3 months in patients not using antihypertensive medication

| Indicators | Group without antihypertensive medication | | | |
| --- | --- | --- | --- | --- |
|  | Model 1 | | Model 2 | |
|  | OR (95% CI) | p | OR (95% CI) | p |
| Clinical factors | － | － | － | － |
| 24-hour SBP SD | 1.38 (1.04–1.83) | 0.024 | 1.46 (1.09–1.95) | 0.010 |
| 24-hour SBP CV | 1.25 (0.94–1.65) | 0.12 | 1.33 (0.99–1.80) | 0.06 |
| 24-hour DBP SD | 1.57 (1.20–2.04) | < 0.001 | 1.57 (1.20–2.06) | 0.001 |
| 24-hour DBP CV | 1.60 (1.20–2.12) | 0.001 | 1.68 (1.25–2.26) | < 0.001 |
| Morning surge | 2.35 (1.21–4.56) | 0.012 | 2.56 (1.28–5.12) | 0.008 |
| Nondipper type | 1.43 (0.62–3.34) | 0.40 | 1.51 (0.64–3.58) | 0.34 |

Multivariable logistic regression analyses were performed to identify indicators (model 1: age, sex, NIHSS score at admission, and each BP parameter using ABPM; model 2: age, sex, variables (except for stroke subtypes) with p values less than 0.20 in the univariate analysis listed in Table 1, and each BP parameter) for poor outcome.

SBP, systolic blood pressure; DBP, diastolic blood pressure; SD, standard deviation; CV, coefficient of variation; OR, odds ratio; CI, confidence interval.
